# Gleanings from Our Exchanges

**Published:** 1872-01-15

**Authors:** 


### Gleanings from Our Exchanges.

### THE INFLUENCE OF A DEFICIENT SUPPLY OF CHLORIDE OF SODIUM UPON THE RISE AND PROGRESS OF CHOLERA.

BY S. WATERMAN, M.D., NEW YORK.

The proposition advanced in my paper before the Academy of Medicine, October 5th, "that the presence of sodium in the air in sufficient quantity is absolutely necessary to life in its many and different forms and conditions; that its antiseptic properties are especially adapted to prevent contamination of air, earth, and water; and that its deficient supply is probably connected with the rise and progress of endemic contagious diseases, such as cholera, typhus and others," is questioned in your December number (p. 444), by my learned friend Dr. Peters.

He states that cholera has been conveyed in a great number of instances across the sodium-salt ocean; that it has broken out in mid-ocean, and has raged in sea-ports where the air is loaded and impregnated with the soda muriate.

To this I deem it necessary to respond, that Dr. Peters finds fault with my conclusions, but leaves my premises uncontradicted. It would be difficult, I think, for any one to deny that sodium in the air in sufficient quantity is absolutely necessary to life in its manifold forms and conditions; nay, it is even very probable, as Prof. Roscoe states in his work on spectral analysis, p. 69, "that these minute particles serve to supply the smaller organized bodies with the salts which larger animals and plants obtain from the ground. If, as is scarcely doubted at the present time, the explanation of the spread of contagious disease is to be sought for in some peculiar contact-action, it is possible that the presence of so antiseptic a substance as chloride-sodium, even in almost infinitely small quantities, may not be without influence upon such occurrences in the atmosphere."

Dr. Peters also does not deny that the sodium-chloride possesses great antiseptic properties, by reason of which contamination of air, earth and water is prevented. Now, it stands to reason that a deficient supply of this antiseptic agent must favor conditions favorable to the development of such contamination, and consequent disease. I think this proposition is logical. That the supply of sodium in the air is subject to daily oscillations is proved by spectroscopic observations, just as the supply of oxygen varies in the animal economy, and is never constant; yet, under normal circumstances, the quantity oscillates only within narrow limits. So also, so long as the supply of the sodium-salt oscillates within defined limits, no perceptible disturbance in the life and health of the animal economy is experienced. But a deficient supply of this antiseptic element for any length of time must be detrimental to organic life, and will favor contaminations of air, earth and water.

Granted, then, that cholera has been conveyed across the sodium-salt ocean. It is most probable that when this disease is once fully developed, the presence of the sodium-salt in the air in sufficient quantity may modify its violence; but we have not claimed that it will prevent its development. Whenever cholera has broken out in mid-ocean, the germ of the disease has been taken on board at the ship's point of departure. Its development in such cases was most undoubtedly favored by crowded steerage, tainted water, tainted food, want of cleanliness and proper ventilation. I deny spontaneous disease in mid-ocean. So also as regards the statement that cholera often rages in sea-ports surrounded with an atmosphere of sodium. No locality is more favorable to the germination of the cholera fungus, or whatever that germ may be, than sea-ports. It finds there its most favorite soil, provided there is, by the short-sightedness and neglect of man, a crowded population, abundance of decaying animal and vegetable matter, narrow, filthy streets, tainted water, obstructed drainage, foul air, want of cleanliness, want of proper food, intemperance, crime of all shades and forms, and in addition, want of intelligent sanitary measures. In vain does Nature pour out its antiseptic spray to protect the lives of improvident man; the reeking foul atmosphere renders the benign influence of the sodium-chloride almost nugatory. When to conditions thus favorable to the development of cholera the supply of the soda-spray becomes deficient from some cause or other, can it be denied that the disease in question will grow in virulency and become boundless in its destructive force?

Dr. Peters further states that cholera has frequently prevailed along the whole Russian or north- coast of the Black Sea, where the so-called salt-limous abound, and sodium-chloride is stored in huge stacks of hundreds of thousands of pounds. No amount of sodium-chloride, stored in one particular locality, is capable of preventing the introduction of cholera, for the simple reason that this salt, in order to reach the circulation, must be in a state of minute division, such only as can be obtained by evaporation from a surface covering two-thirds of our globe, and by the action of storms and winds over its surface. The thoughtful physicist will at once recognize that these fifty thousand Russian cartmen, with their million of oxen and a quarter of a million of carts, may become excellent agents to carry along the cholera on the highway of commerce, in spite of their loads of salt.

The Russian cartmen are none of the cleanest; they subsist on inferior food, are fond of alcoholic drink, in which they liberally indulge, and on their long journey they and their animals are exposed to exhausting influences. *But yet, true cholera has never originated in these salt-limous.* Whenever the cholera has reached these regions, it was in the line of its migration, and had ere this obtained its characteristic destructiveness, which could not be arrested by the tons of stored-up salts of which the air may have been defectively supplied. On the contrary, the mixed crowd of men and beasts, promiscuously thrown together under the circumstances above enumerated, was well calculated to give the disease a high degree of virulence. In addition to this, it must not be forgotten that this vast deposit of salt in these Russian salt-limous results from the evaporation of sea water in swampy grounds, favoring the development of malaria and mephitic gases during the hot months of June and July, just as our sea-marshes do along the Atlantic coast, where, in spite of the modifying antiseptic influences of the sodium spray, intermittent fevers abound.

It is to be regretted that in 1817, when the cholera first appeared in Jessore, not far from Calcutta, after an uncommonly long season of rain, spectroscopic analysis was unknown, and observations were not instituted registering the daily supply of sodium in the air. Such observations alone are destined to offer a satisfactory solution to my proposition, which, notwithstanding the formidable array of figures presented by my learned friend, I shall consider unshaken and not disproved.—*N. Y. Medical Record.*

Clinical Treatment of Pneumonia.—Prof. Lebert, of Breslau (*Transln. in the Clinic*), says that the ground basis of the treatment of pneumonia, so long as its peculiar manifestations exist in moderate grade, threatening no immediate danger, should be eminently expectant, chiefly only dietetic-hygienic. He has treated more than forty cases during the past year, for the most part negatively, and finds that patients feel subjectively much better, by this method, than under the former plans with antimony, digitalis, veratrine, etc. They are spared, too, in this way, the temporary disease caused by the remedy itself. Warm bed-covers are condemned. In Breslau it is the custom to sleep between feather-beds. These have been thrust out in his clinic, and patients become accustomed to the frequent opening of the windows by day, even in the coldest weather of winter. Notwithstanding the lessened appetite, the patient, even in still increasing fever, is not to be allowed to fast entirely, which is too generally the rule; light gruels of barley and oat-meal, in violent thirst in greater quantities, with nourishing and alleviating drinks. In intense fever, hard pulse, if no diarrhœa be present, acid drinks are most agreeable; the best is cold lemonade, only moderately sweetened and prepared from fresh lemons. Mixtures of fruit syrups, raspberry-juice, etc., are occasionally temporarily agreeable, but are usually not long palatable. Many patients prefer warm drinks, and they suit better when there is inclination to racking cough or diarrhœa. Common tea, from a teaspoonful to three-quarters of a cup, is a good drink in relieving irritation. Seltzer-water with milk is refreshing when the fever begins to diminish and blood no longnr presents in the sputa. When the appetite is very poor, infusions of quassia and decoctions of Iceland moss are of value. In very reduced patients, in typhoid conditions of the disease, he generally adds to the teas or other drinks a teaspoonful or two of ether syrup to the glass or dessertspoonful of Rhine, red or Hungarian wine. Under such conditions the combination of wine and lemonade is excellent.—*N. Y. Med. Record.*

Removal of the Kidney during Life.—Prof. Simon, of Heidelberg (Schmidt's *Yahrbucher*, Bd. 151, Nr. 7, 1871; from the *Deutsche Klin.*, xv. 137, 1870), reports that he removed the left kidney during life from a woman twenty-six years of age, under the following circumstances: A year and a half before, ovariotomy had been performed, when, in consequence of the firm adhesion of the tumor to the adjacent organs, it was necessary to remove the uterus and to divide the ureter; the divided end of the ureter becoming adherent to the abdominal wall just above the symphysis pubis, the urine from the left kidney afterwards flowed through this fistulous orifice. An attempt to establish a communication between the ureter and the blader, and in this way to close the fistulous orifice, was abandoned in consequence of the patient having been rendered seriously ill by it. It was found equally impossible, for the same reason, to produce an artificial closure of the ureter and atrophy of the kidney. It was therefore determined to extirpate the organ from behind the peritoneum which was accordingly done. The patient bore the operation very well, and was able to leave her bed at the end of six weeks. The ligature which had been placed around the pedicle did not come away for six months; after which a great improvement in her health was observed.

Another case of extirpation of the kidney (same journal, from *Wurtemb. Corr.*, Bl. lxi. 14, 1871) is reported by Dr. Linser. In this case the patient was a soldier who had been wounded in the left lumbar region. Bloody urine flowed through the wound at first, and subsequently pus mixed with blood. An incision was made from the twelfth rib to the crest of the ilium; but the kidney was so adherent to its capsule that it was necessary to remove part by the scissors. The patient sank eight hours after the operation.—*Philadelphia Medical Times.*

The Treatment of Small-pox.—Dr. Alexander Collie, the resident medical officer at the Homerton Fever Hospital, says that treatment in the mild variety of variola is unnecessary, and in the black small-pox useless. In the confluent form, however, treatment is of the greatest importance, and the result of the case will sometimes be determined by it. The room in which the patient is placed should be thoroughly ventilated, the windows being kept open even in winter. If possible, there should be two beds in his room, in order that he may be changed from one to the other. He should be allowed a highly nutritious diet, consisting of milk, beef-tea, eggs beaten up with whiskey, tapioca. Cold water will be found most efficacious in relieving thirst, and the prejudice of the patient's friends should not be allowed to interfere with its administration. Effervescing drinks and lemonade may also be allowed. For heat of skin the patient may be sponged with cold water two or three times daily. If there be much restlessness or sleeplessness, the following repeated in half an hour, if needed, will be found of great service: tincture of opium, fifteen minums; spirit of ether, fifteen minums; camphor water, one ounce; and if this fails, stimulants may be given. For the soreness of throat, oleaginous or mucilaginous drinks may be given, and the following has also been found beneficial: tincture of iron and glycerine, of each, thirty minums three times a day. If laryngitis occur, a large linseed poultice should be supplied round the throat, and the temperature of the room should be raised, and rendered moist by means of steam. All depressing remedies should be avoided, and tracheotomy should be performed whenever there is much interference with the respiration. If the patient becomes delirious, it is of the utmost importance that he should be treated with patience, gentleness and firmness, and that no measures of restraint should be employed. Nothing has been found absolutely preventive of pitting. Common olive oil may be used for this purpose in preference to applications which are more or less irritating. If diarrhœa occur a mixture containing laudanum and sulphuric acid may be given.

In regard to the time when a small-pox patient may be considered free of danger to his neighbors, Dr. Collie says that this cannot be until all the products of disease are removed from his body, and until he presents all the ordinary indications of health; such as a normal temperature, a quiet pulse, a clean tongue, a clear mind, etc.—*Philadelphia Medical Times.*

Excision of Cancer of the Œsophagus.—The last number of the *Archiv fur Klinische Chirurgie* contains a paper by Dr. Billroth, in which he proposes, in cases of carcinomatous stricture of the œsophagus occurring in an accessible situation, to cut out, through its whole circumference, that portion of the tube which contains the disease. He has not yet performed this operation on the human subject; but he is led to believe in its practicability, first, from the occasional restoration of the urethral canal after its division, and secondly by the result of an experiment which he performed on a dog. He cut out about an inch and a quarter of the animal's œsophagus, fastened the lower end to the edge of the wound by two sutures, and fed the dog with milk through an œsophageal tube passed through the mouth into the stomach. The sutures were removed about a week after the operation. There was considerable mucous discharge from the wound. The external wound gradually contracted, and the discharge diminished. About ten weeks after the operation the external opening was completely closed. Bougies were frequently introduced so as to dilate the cicatrix, and the dog gradually regained the power of eating flesh, potatoes, etc., and swallowed them with ease. Three months after the operation the animal was killed. The œsophagus presented a simple annular cicatrix, scarcely half a line wide; the tube was completely pervious.—*Ibid.*

Scarlatina.—Dr. Richard Inglis, of Detroit, Mich., (*Review of Med.*) presents an interesting paper on scarlatina, in which the early history of this malady is noted. Ingrassia, of Naples, was about the first to describe it accurately; it was called by him, in A.D. 1500, *rossalia;* in 1565 it was epidemic in Paris, and was named by Ballonias, *rubiola;* he carefully distinguishes it from measles, *morbille.* In 1580 it was known in Spain as the *garotillo;* in 1680, in Italy, as *morbillo ignei.* It was not recognized in England as a disease differing from measles until 1650. Probably the first record of its appearance in this country is an account of an epidemic which broke out in Kingston township, fifty miles eastward from Boston, in 1735. Dr. Douglas, of Boston, has left a record of it under the title of the "Practical History of a new Epidemical Miliary Fever with an Angina Ulcusculosa."—*Ibid.*

